# Dependence of Tensile Properties and Fracture Behaviors on the Fractions of Continuous and Discontinuous Precipitates in Peak-Aged AZ80A Magnesium Alloy

**DOI:** 10.3390/ma16134546

**Published:** 2023-06-23

**Authors:** Kelong Zhang, Huizhong Li, Xiaopeng Liang, Zhi Chen, Hui Tao, Yixuan Che, Li Li, Zixiang Luo, Qinghuan Huo

**Affiliations:** 1 School of Materials Science and Engineering, Central South University, Changsha 410083, Chinaxpliang@csu.edu.cn (X.L.);; 2 State Key Laboratory of Powder Metallurgy, Central South University, Changsha 410083, China; 3 Key Laboratory of Nonferrous Metal Materials Science and Engineering, Ministry of Education, Central South University, Changsha 410083, China

**Keywords:** magnesium alloy, continuous precipitation, tensile properties, fracture behavior, precipitation-strengthening

## Abstract

After T5 (forging + aging) and different T6 (forging + solution + aging) heat treatments, the AZ80A Mg alloys exhibited microstructures with different fractions of continuous precipitate (CP) regions and discontinuous precipitate (DP) regions. The effects of the fractions of DP regions and CP regions on the tensile properties and fracture behaviors were investigated using microstructural characterizations and analysis. The results showed that increasing the fraction of DP regions enhanced the yield strength and tensile strength at room temperature. However, at the same high temperature, increasing the fractions of DP regions improved the elongation but deteriorated the tensile strength significantly. The different resultant tensile properties at different temperatures were caused by the different precipitation-strengthening effects in the CP and DP regions. The strengthening contribution of the DP regions was more effective at room temperature but became inferior to the effect brought about by the CP regions at high temperatures. Micro-cracks were usually initiated and propagated in the CP regions at room temperature. At high temperatures, however, micro-voids formed more easily in the DP regions, and the fracture path preferred to locate there.

## 1. Introduction

Benefiting from low density, high specific strength, excellent hot working performance, outstanding fracture toughness, and relatively low cost [1,2,3,4,5,6], Mg-Al series alloys are becoming more and more attractive in the transportation and oil industries where weight reduction, fuel economy, and environmental friendliness are increasingly demanded [7]. Currently, many automotive components, including instrument panels and steering wheel armatures, are manufactured with conventional magnesium alloys (i.e., AM and AZ alloys) [8]. However, their relatively low strength, especially at high temperatures, becomes a bottleneck problem for further applications. Therefore, increasing the strength at high operating temperatures has a significant meaning for expanding the application of magnesium alloys.

In recent years a great deal of effort has been made to overcome this bottleneck problem. Recent studies have shown that the high-temperature mechanical properties of wrought Mg-Al based alloys, such as AZ91 [9,10,11], AZ80 [12], and AZ61 [13], can be improved by the trace addition of rare earth elements (REs). Because rare earth elements are non-renewable resources, and the addition of rare earth elements significantly increases the cost of the material, a more economical way of providing elevated-temperature strength of Mg-Al series alloys is urgently needed.

The improvement in high-temperature mechanical properties by the addition of rare earth elements is often attributed to the formation of thermally stable Al-RE phases [14,15]. At the same time, the amount of discontinuous precipitates—one kind of β-Mg_17_Al_12_ phase—obviously decreases, which may also play an important role. In Mg-Al-based alloys, the β phases in Mg-Al series alloys are precipitated competitively by continuous precipitation (CP) [16,17,18,19] and discontinuous precipitation (DP) during aging [20,21,22,23]. Because the continuous precipitates (CPs) induced by CP and the discontinuous precipitates (DPs) conducted by DP have great differences in terms of morphology and distribution, they are different in their precipitation-strengthening effect on the alloys [24]. Furthermore, their precipitation-strengthening effects are likely to vary with temperature because the deformation mechanisms of magnesium alloys at room temperature are also different from those at high temperatures [25,26,27]. Therefore, revealing and comparing the precipitation-strengthening effects of continuous and discontinuous precipitates at different temperatures has great significance, and controlling their ratio is a feasible method to improve the mechanical properties of the alloy at room and high temperatures.

Researchers control the ratio of CP- and DP-region fractions using many methods, including adjusting the aging temperature and time [24,28], grain refinement [29], micro alloying [22,30], and pre-twinning deformation [31]. However, other factors affecting the mechanical properties are also induced, such as the sizes and distribution of the precipitates, grain size, new strengthening phases, and twinning. Jun [32] reported that the strength of the cast AZ91 magnesium alloy increases as the volume fraction of discontinuous precipitates (DPs) at room temperature increases. However, the different volume fractions were obtained by varying the aging time; therefore, it is reasonable to doubt that the results are induced by an increase in the whole volume fraction of the Mg_17_Al_12_ phase rather than the volume fraction of the DP regions. Moreover, the effect of the DP region fraction on the high-temperature tensile properties has not been referred to. It is found that the aged AM-SC1 magnesium alloy shows an obviously higher yield strength, ultimate tensile strength, comparable elongation, and a higher rotary bending fatigue strength after solution treatment at higher temperatures [33]. However, the effect of the solution temperature on DP and CP and the resultant mechanical properties of the AZ80A magnesium alloy are seldom reported. Therefore, it is of great significance to study the influence of solution temperature on the aging microstructure and mechanical properties.

This paper aims to study the effect of DP- and CP-region fractions on the tensile properties and fracture behavior of the AZ80A magnesium alloy at different temperatures. Different DP- and CP-region fractions were obtained from T5 and T6 heat treatments with different solution temperatures and the same aging temperature. In order to study the mechanism of the solution-temperature effect on the aging microstructure and the effects of DPs and CPs on the mechanical properties, the microstructures before and after tensile testing were studied using optical microscopes, scanning electron microscopes, and transmission electron microscopes.

## 2. Experimental Methods

A semi-continuous cast ingot of the AZ80A magnesium alloy with a diameter of 300 mm and a length of 650 mm was prepared. Then the casting ingot was homogenized at 410 °C for 24 h and forged by three passes on a 40,000 kN forging machine immediately. After air cooling, a billet with a diameter of 730 mm, a length of 110 mm, a chemical composition (wt.%) of 8.06 Al, 0.62 Zn, 0.23 Mn, and balanced Mg was obtained.

Rectangular “sheets” with a size of 110 mm × 12 mm × 3 mm were cut from the billet and divided into four parts for different heat treatments, as shown in Table 1. One of them was aged at 175 °C for different lengths of time; the other three parts were solution-treated at 420 °C, 450 °C, and 480 °C for 30 min, followed by aging at 175 °C for different lengths of time. Rectangular specimens for hardness testing with a size of 8 mm × 10 mm × 3 mm and flat tensile test specimens with a gauge length of 25 mm, a width of 3 mm, and a thickness of 3 mm were cut from those heat-treated slabs. Tensile experiments were carried out on an tensile test machine (MTS810, MTS, Eden Prairie, MN, USA) at room temperature, 100 °C, 125 °C, 150 °C, and 175 °C. Hardness testing was conducted with a Vickers hardness machine (HVS 1000S, HY, Laizhou, China) with a load of 4.98 N and a loading time of 15 s.

Optical microscopy (OM), scanning electron microscopy (SEM, SIRION200, FEI, Hillsboro, OR, USA), and transmission electron microscopy (TEM, Tecnai G2 20 ST, 200 KV, FEI, Hillsboro, OR, USA) were used to investigate the microstructures. Specimens for OM and SEM observation were etched with a solution composed of 0.5 vol.% nitric acid and 99.5 vol.% alcohol. Thin foils for TEM observation were prepared by the ion beam thinning technique. The fractions of DP and CP regions were calculated with the IPP (Image-Pro Plus 6.0.0.260) software from at least five SEM pictures.

## 3. Results

### 3.1. Microstructures of Solid Solution Treated Specimens

**Figure 1:**
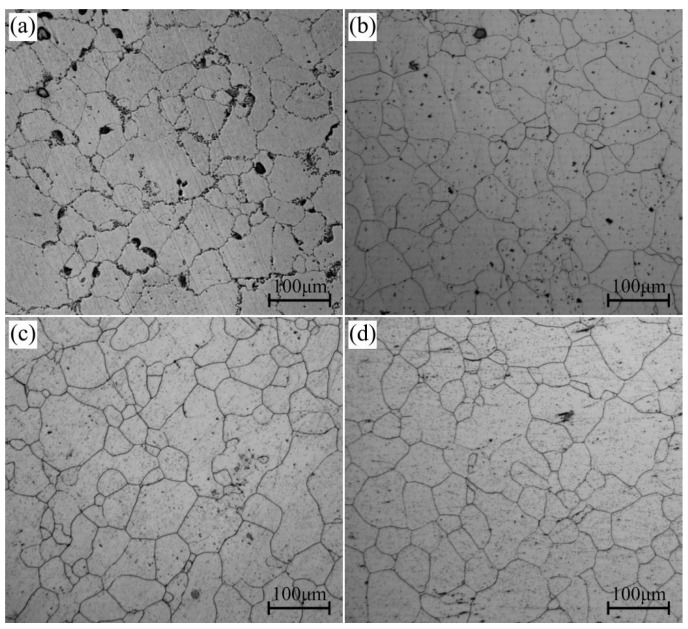
Microstructures of specimens after (**a**) forging and (**b**–**d**) solid solution treatment at 420 °C, 450 °C, and 480 °C, respectively.

Figure 1 shows the OM microstructures of the AZ80A magnesium alloy at the forged stage and solid solution-treated state. At the forged stage, the grain boundaries are serrated, and certain small and thin cellular structures emerge along the grain boundaries, as shown in Figure 1a. After 30 min of solid solution treatment at 420 °C, 450 °C, and 480 °C, tiny and thin cellular structures vanish, and the serrated boundaries are replaced by flat boundaries. Significantly, the grain size has no obvious change after solid solution treatment and even stays stable with the increase in solution temperature, as shown in Figure 1b–d.

### 3.2. Age-Hardening Behavior

**Figure 2:**
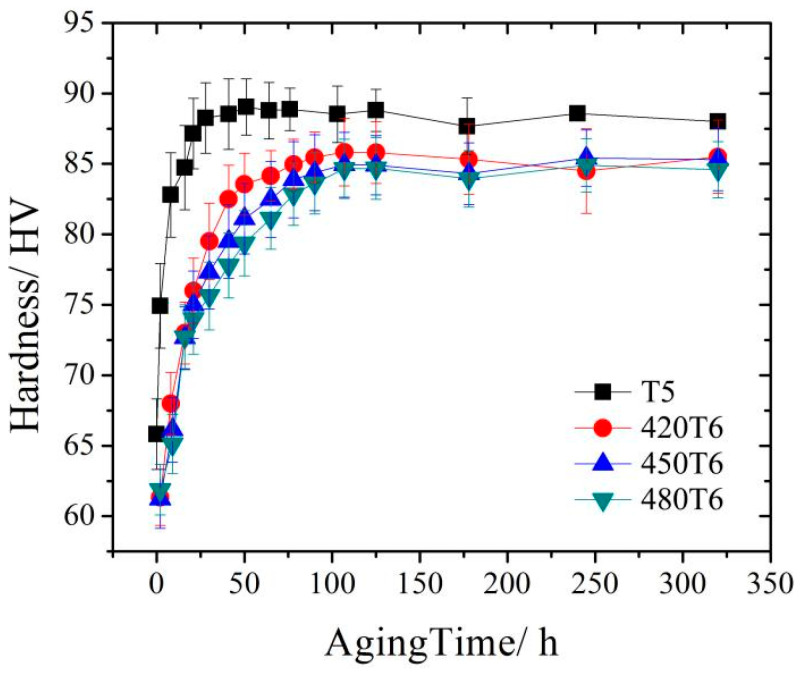
Age-hardening curve of the AZ80A magnesium alloy after forging and solid solute heat treatment at different temperatures.

Figure 2 shows the age-hardening behavior of the forged and solid solution-treated AZ80A magnesium alloy specimens at 175 °C. The hardness of the forged specimens increases quickly with the increasing aging time and reaches its peak value at 50 h, followed by a clear declining trend. For the specimens of solid solution treatment at different temperatures, their hardness increases rapidly at the beginning of the aging process, and their peak hardness arrives simultaneously at 100 h without noticeable over-aging phenomena at the end of the aging procedure. Apparently, not only did the peak hardness of the forged specimens occur earlier than that of the solid solution-treated specimens, but the former also has a substantially higher peak value than the latter. Furthermore, as the solid solution temperature rises, the peak-aging hardness falls significantly.

### 3.3. Microstructures of Aged Specimens

**Figure 3:**
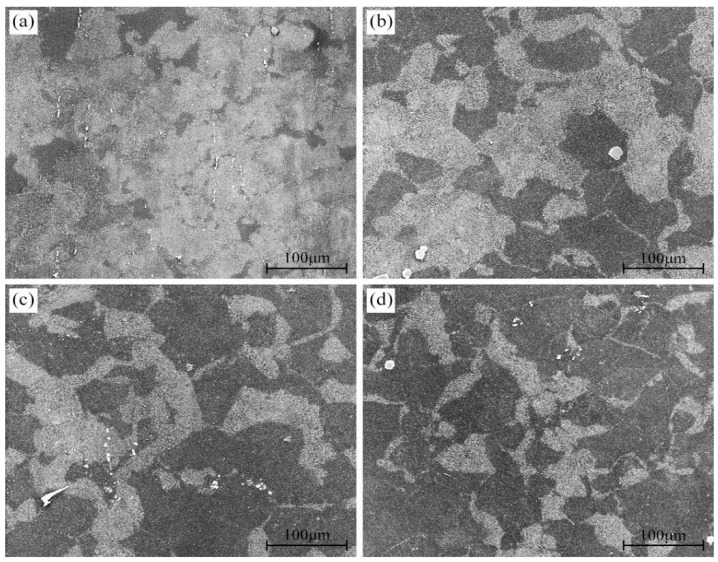
Microstructures of the AZ80A alloy at different peak-aged states: T5 (**a**); 420T6 (**b**); 450T6 (**c**); 480T6 (**d**).

Figure 3 shows the SEM microstructures of different peak-aged AZ80A magnesium alloy specimens. The bright and dark regions of the SEM microstructures have been confirmed to be DP and CP regions in our previous study [24]. The size of the DP regions in T5 peak-aged specimens is much larger than that of T6 peak-aged specimens. Moreover, with the increase in solid solution temperature, the size of the DP regions decreases continuously.

**Figure 4:**
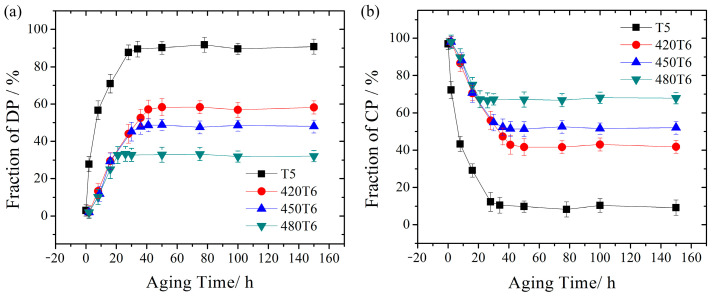
Fraction variation with the aging time: (**a**) DP region and (**b**) CP region.

In order to figure out the variation of different precipitated phase regions, the changes in the fraction of DP and CP regions with the increase in aging time in different heat-treated specimens are calculated, as shown in Figure 4. During the T5 aging process, the fraction of the DP regions increases at first as the aging time increases. After aging for 28 h, it reaches a maximum value and retains it dynamically for the following period of time. The value of the DP fraction during the T5 aging process increases faster than it does during the T6 aging process. Besides, the maximum value of the DP fraction during the T5 process is much larger than during the T6 process, as shown in Figure 4a. During the T6 aging process, the DP-region fraction of the specimens with different solution temperatures almost increases at the same rate. When the sample was aged after the solution treatment at 420 °C, the DP-region fraction increased to 57% at 50 h and then stopped increasing. When increasing the solution temperature to 450 °C and 480 °C, the increasing rate of the DP-region fraction remains stable. However, the increasing times are shortened gradually, leading to a decrease in the final DP-region fraction of 48% and 32%, and an increase in the final CP-region fraction of 52% and 68%, as shown in Figure 4b. It can be inferred that a high-temperature solid solution treatment benefits CP in its competition with DP. 

### 3.4. Tensile Properties

#### 3.4.1. Tensile Properties at Room Temperature

**Figure 5:**
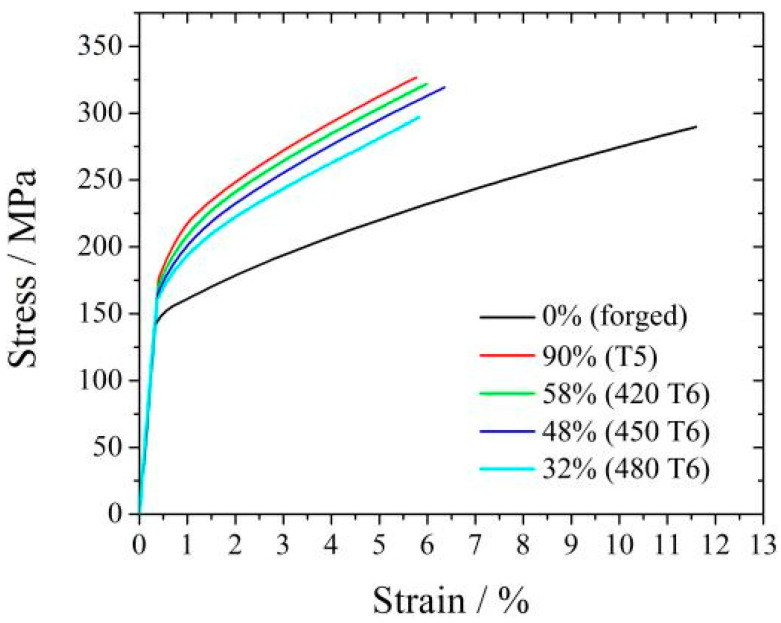
Tensile stress–strain curves of four samples. The DP-region fraction is also marked.

The stress–strain curves of different peak-aged specimens tensile-tested at room temperature are shown in Figure 5, and tensile mechanical properties are described in Table 2. The yield strength, tensile strength, and elongation of the T5 peak-aged specimen with a DP-region fraction of 90% are 218 MPa, 327 MPa, and 4.1%, respectively. When the alloy is T6 peak-aged, the yield strength and tensile strength weaken and decrease continuously as the solid solution temperature increases. When the solution temperature increases to 480 °C, the yield strength and tensile strength decrease to 185 MPa and 296 MPa, respectively. However, the elongation of different peak-aged specimens is very close. Meanwhile, the yield strength and tensile strength decrease as the DP-region fraction decreases. Therefore, DP is more beneficial in improving the tensile properties at room temperature than CP.

#### 3.4.2. Tensile Properties at High Temperature

**Figure 6:**
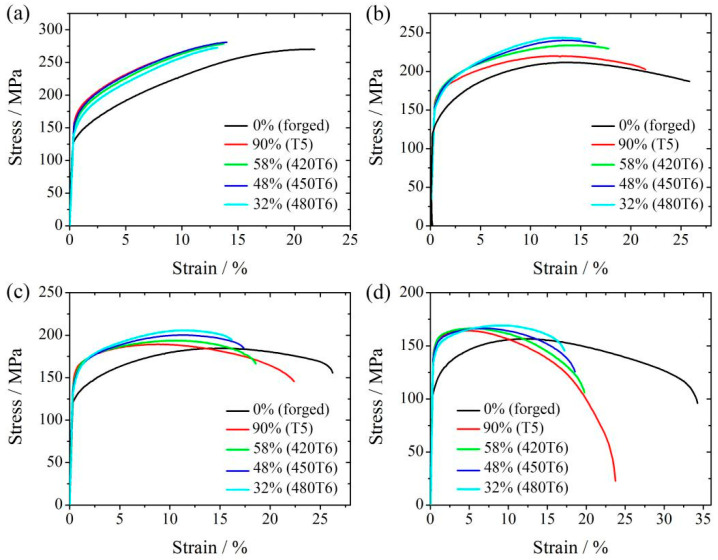
The tensile stress–strain curves of peak-aged specimens tensile-tested at 100 °C (**a**), 125 °C (**b**), 150 °C (**c**), 175 °C (**d**). The DP-region fraction is also marked.

Figure 6 shows the tensile stress–strain curve of the peak-aged specimens tensile-tested in the temperature range of 100 °C–175 °C, and the tensile mechanical properties are summarized in Table 3. With the tensile temperature increasing, all specimens’ yield strength and tensile strength decrease, but their elongation increases. At 100 °C, the yield strength still decreases with the DP fraction, but the tensile strength and elongation change slightly. When the tensile test is carried out above 100 °C, the variations in the yield strength and the elongation with the DP region fraction follow the same trend. However, the tensile strength increases as the DP-region fraction decreases. The increasing tendency becomes most apparent when the test temperature is 125 °C. The yield strength of the T5 peak-aged specimen with a DP-region fraction of 90% (169 MPa) is 14 MPa higher than that of the 480T6 peak-aged specimen with a DP-region fraction of 32% (155 MPa), but the tensile strength of the latter (244 MPa) is 24 MPa higher than the former (220 MPa), as shown in Figure 6b. Therefore, it can be seen that a high DP-region fraction is harmful to the tensile strength at high temperatures, whereas a high CP-region fraction benefits the strength.

### 3.5. Fracture Morphology

**Figure 7:**
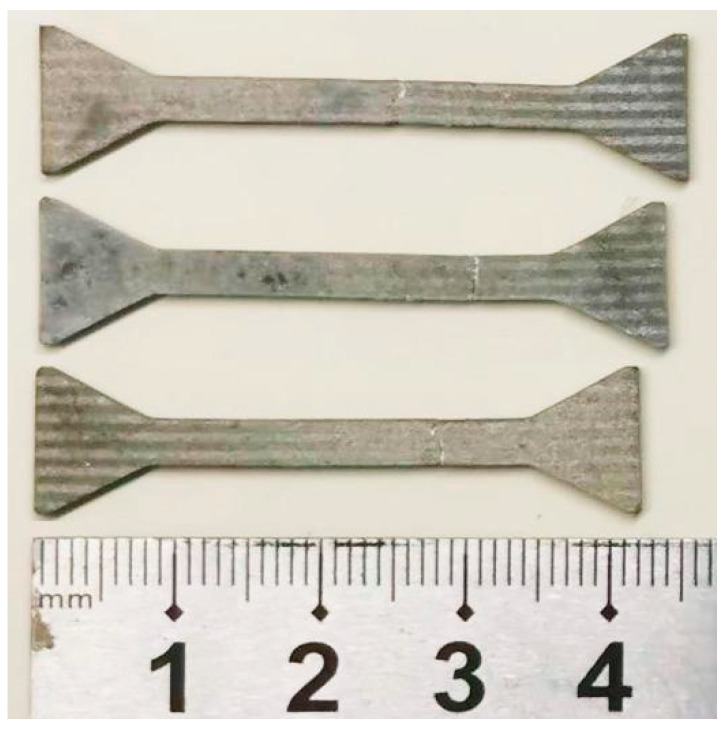
The 450T6 specimens with a 48% DP-region fraction tensile-tested until failure at different temperatures.

**Figure 8:**
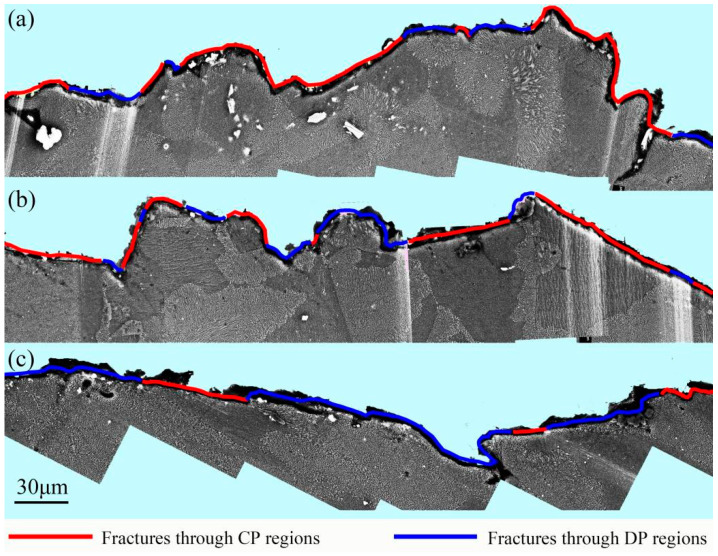
Crack propagation behavior in the 450T6 specimens with a 48% DP-region fraction tensile-tested at room temperature (**a**), 100 °C (**b**), and 175 °C (**c**).

**Figure 9:**
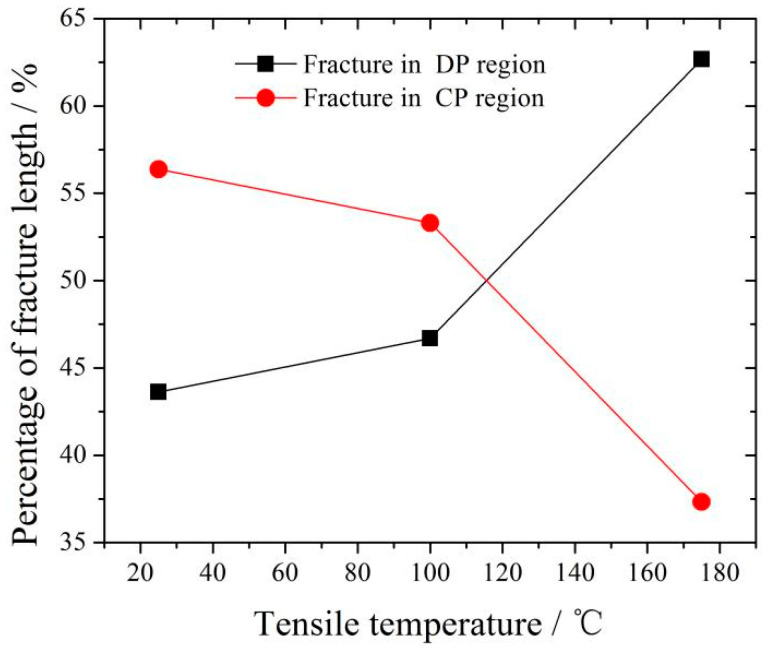
Percentage of fracture in DP and CP regions of the 450T6 specimens with a 48% DP-region tensile-tested at room temperature, 100 °C, and 175 °C.

Figure 7 shows the 450T6 specimens with a 48% DP-region fraction after tensile testing until failure at different temperatures. It shows that the extension increases with the tensile temperature. Figure 8 presents the SEM images of the longitudinal section of the fracture in the 450T6 specimens subjected to tensile tests at different temperatures. The crack-propagation path information is obtained from the longitudinal section of the fracture. The percentage of the path located in DP and CP regions is calculated and plotted in Figure 9. At room temperature, the path located in CP regions is much more prevalent than in DP regions, as shown in Figure 8a. With the increase in the tensile temperature, the total length of the path diminishes in CP regions but augments in DP regions, as shown in Figure 8b,c. At 100 °C, the percentage of the path in DP regions increases to 46.7%, almost equivalent to the DP-region fraction of the specimen. When the tensile temperature rises to 175 °C, the percentage of the path in DP regions exceeds that in CP regions, as shown in Figure 9.

**Figure 10:**
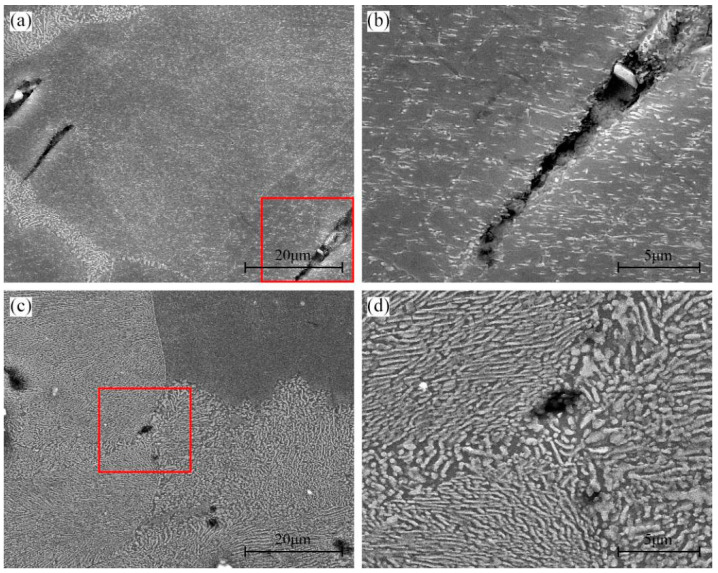
Crack initiation in the 450T6 specimens with a 48% DP-region fraction tensile-tested at room temperature (**a**,**b**) and 175 °C (**c**,**d**). (**c**) and (**d**) are the magnified region of the red frame in (**a**) and (**c**), respectively.

Figure 10 shows the micro-cracks at the initiation stage in specimens with 48% DP regions tensile-tested at room temperature and 175 °C. When the specimen was tensile-tested at room temperature, some micro-cracks appeared in the CP region, as shown in Figure 10a,b. After tensile testing at 175 °C, micro-cracks are hardly seen in CP regions, while many micro-voids form in DP regions, as shown in Figure 10c,d.

**Figure 11:**
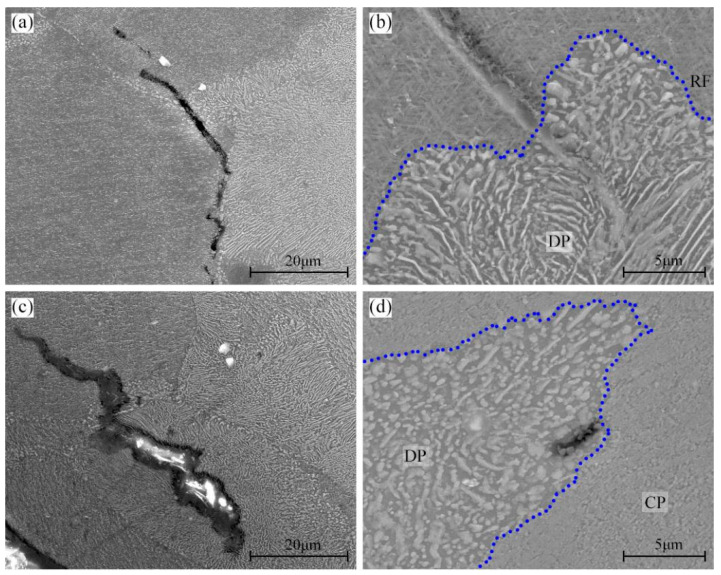
Crack propagation behavior in the 450T6 specimens with a 48% DP-region fraction tensile-tested at room temperature (**a**), 100 °C (**b**,**c**), and 175 °C (**d**). Blue line is the RF, one of the boundary of DP regions.

Figure 11 shows the crack propagation behavior in the specimens with 48% DP regions tensile-tested at room temperature, 100 °C, and 175 °C. At room temperature, when the growing cracks in CP regions encounter a DP region, it is too hard for them to invade the DP region and they usually alter their propagation direction to avoid the DP regions, as shown in Figure 11a. At 100 °C, not only can the growing cracks in CP regions grow directly into the DP regions, but also, those in DP regions can propagate smoothly into the CP region, as shown in Figure 11b,c. When the specimen was tensile-tested at 175 °C, the cavities in DP regions develop into micro-cracks. However, the crack propagation is hindered by the CP regions, as shown in Figure 11d.

**Figure 12:**
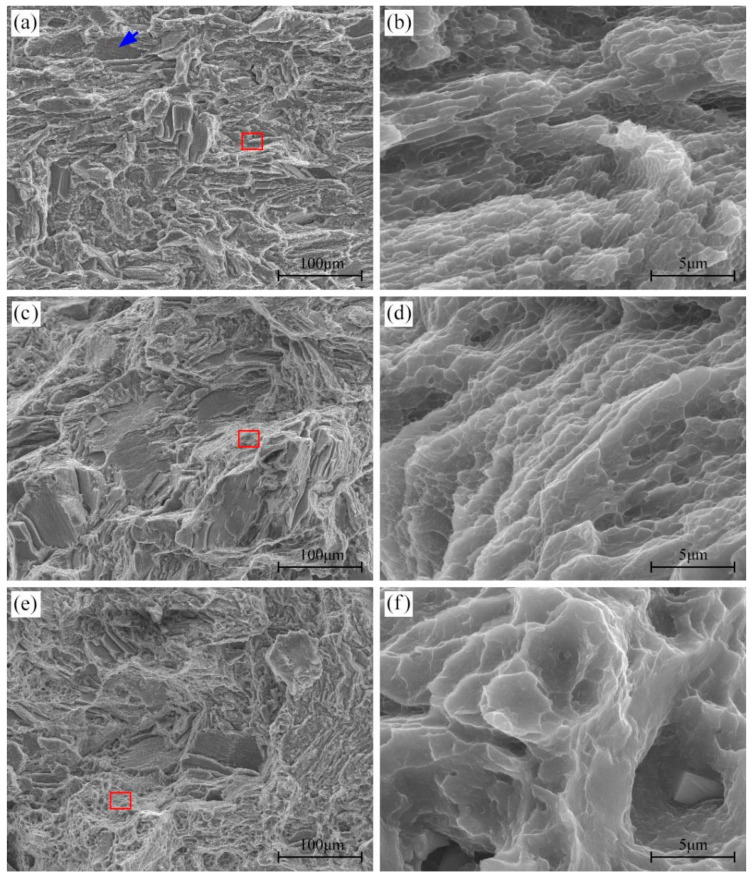
Fracture morphology of 450T6 specimens containing a 48% DP-region fraction after tensile testing at room temperature (**a**,**b**), 100 °C (**c**,**d**), and 175 °C (**e**,**f**). The blue arrow indicates the quasi-cleavage planes. (**b**), (**d**) and (**f**) are the magnified regions of the red frame in (**a**), (**c**) and (**e**).

Figure 12 shows the fracture morphology of 450T6 specimens with a 48% DP fraction subjected to tensile tests at different temperatures. The fracture surface obtained after tensile testing at room temperature exhibits quasi-cleavage planes (indicated by blue arrows) and tearing ridges, as shown in Figure 12a. In addition, regions adjacent to the tearing ridges are populated with many small dimples, as shown in Figure 12b. It implies that the 450T6 specimens undergo a quasi-cleavage mode of failure when they are tensile-tested at room temperature. As the tensile temperature rises to 175 °C, quasi-cleavage planes and tearing ridges persist, but the amount of quasi-cleavage planes gradually diminishes, and the dimples progressively enlarge with the increasing tensile temperature, as shown in Figure 12c–f. It suggests that quasi-cleavage failure still operates at a tensile temperature as high as 175 °C, but the propensity for ductile fracture also increases.

**Figure 13:**
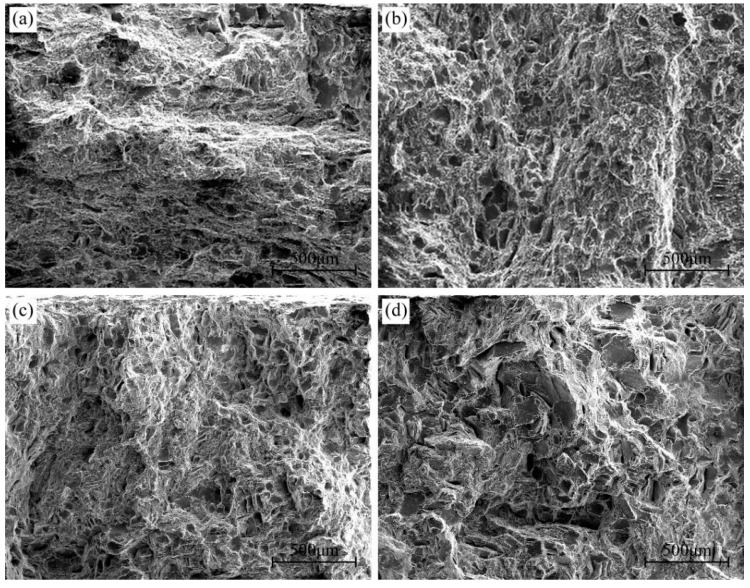
Fracture morphology of different peak-aged specimens containing 90% (T5) (**a**), 58% (420T6) (**b**), 48% (450T6) (**c**), and 32% (480T6) (**d**) DP-region fractions after tensile testing at 125 °C.

Figure 13 depicts the fracture morphology of different peak-aged specimens after tensile testing at 125 °C. Quasi-cleavage planes and tearing ridges coexist on each fracture surface. However, the quantity and dimension of quasi-cleavage planes augment as the DP-region fraction decreases. It indicates that all specimens fracture in the quasi-cleavage mode at 125 °C, and the propensity for brittle fracture intensifies as the DP-region fraction increases.

### 3.6. TEM Microstructures near the Tensile Fracture

**Figure 14:**
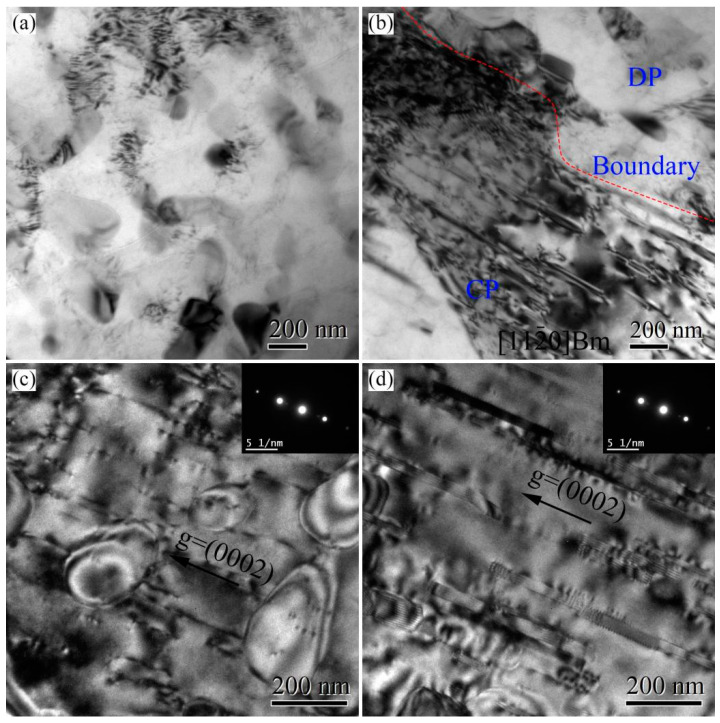
TEM bright fields of DP and CP regions near the fracture of the peak-aged AZ80A magnesium alloy after tensile testing at different temperatures: (**a**,**b**) room temperature; (**c**,**d**) 150 °C.

Figure 14 shows the TEM microstructures near the fracture of the tensile-tested 420T6 specimens. After tensile testing at room temperature and 150 °C, many dislocations occur and interact with precipitates. At room temperature, dislocations in DP regions are blocked by elliptical precipitates, resulting in dislocation clusters, as shown in Figure 14a. In CP regions, dislocations are stacked near the grain boundary, but the low dislocation density is exhibited far from the grain boundary, as shown in Figure 14b. As is well known, only the basal slip system can be activated in magnesium alloys during room temperature deformation. Therefore, those dislocations in Figure 14a,b are basal dislocations. Two-beam diffraction for the specimen tensile-tested at 150 °C is obtained under the diffraction vector of g = [0002] along the [112(_)0] zone axis. According to the visibility criterion of dislocations (g∙b ≠ 0, b: Burgers vector) [34], all <a> dislocations on the basal planes and the prismatic planes become invisible under this condition. The observed dislocations in Figure 14c,d are all <c> components, which belong to the pyramidal <c + a> slip. Figure 14c shows that many dislocations in DP regions are dispersed in the matrix, and some are impeded by elliptical precipitates. Meanwhile, some dislocations are also generated inside the elliptical precipitates, indicating that the precipitates also undergo plastic deformation. In CP regions, most of the dislocations are attached to the plate precipitates, as shown in Figure 14d. This suggests that the non-basal dislocations are strongly hindered by the plate precipitates during the slip process.

## 4. Discussion

### 4.1. Effect of Solid Solution Treatment on the Aged Microstructures

When the alloy is peak-aged at 175 °C, the fraction of the DP region in the T5 specimen increases much faster and reaches a much higher final value than that in 420T6 specimens. A similar phenomenon has been reported in the literature [35]. The reason is generally attributed to the serrated grain boundaries in the forged state, which provide far more nucleation sites for DP precipitates.

When the solid solution temperature increases to 450 °C and 480 °C, the fraction of DP regions decreases, and the fraction of CP regions increases. This results from the constant growth rate and decrease in the growth time of DP regions. The growth time is the crucial factor in this phenomenon. As the solution temperature increases, the equilibrium vacancy concentration increases. After water cooling, a higher supersaturated vacancy concentration remains. It would accelerate the CP reaction in the following aging process because the CP reaction is controlled by volume diffusion, and a higher vacancy concentration increases the diffusion coefficient [24]. According to the DP reaction mechanism [23,28], the DP reaction only stops when the Al atoms are exhausted by the CP reaction or when the CP precipitates grow large enough to impede the movement of the DP reaction front. Therefore, when the solution temperature increases, the CP reaction is accelerated, and the DP reaction is terminated earlier, leading to a smaller final fraction of DP regions.

### 4.2. Dependence of Mechanical Properties on the Fraction of the DP Region

#### 4.2.1. Room-Temperature Strength

Increasing the fraction of DP regions in the peak-aged AZ80A magnesium alloy constantly increases the peak hardness, yield strength, and tensile strength at room temperature. In magnesium alloys, except for the basal slip system, few slip systems can be activated at room temperature [25]. As mentioned in our previous study, the precipitates in CP regions are dominated by plate phases with the thin plate parallel to the basal plane of the matrix. In comparison, the primary precipitates of elliptical and lamellar phases in DP regions are randomly distributed. As a result, the basal slipping is hindered much more strongly by precipitates in DP regions than that in CP regions, even though precipitates in CP regions are much smaller and denser than those in DP regions, which is supported by the dislocation interaction shown in Figure 14a,b. Therefore, the precipitation-strengthening effect of DP regions is better than that of CP regions at ambient temperature. Logically, increasing DP regions helps to improve the strength of the AZ80A magnesium alloy.

#### 4.2.2. High-Temperature Strength

When the tensile temperature is above 100 °C, the increase in yield strength, as the DP-region fraction increases, is decelerated, and the tensile strength even decreases as DP regions increase. As is well known, non-basal slipping can be activated at high temperatures, and the percentage of non-basal dislocations increases with the deformation temperature [25]. The plate phases, compared with the elliptical and lamellar phases, significantly enhance the hindering effect of non-basal slipping, as shown in Figure 14c,d. This is because the plate phases have a uniform habit plane, effectively impeding non-basal slipping. Moreover, the precipitates in CP regions are much smaller and denser than those in DP regions, which indicates a more substantial hindering effect on dislocations of the precipitates in CP regions than on those in DP regions, according to the Orowan mechanism [24,36,37]. Therefore, the precipitation-strengthening of CP regions is much more pronounced than that of DP regions at high temperatures. It explains why the tensile strength increases with the increase in the fraction of CP regions and the decrease in the fraction of DP regions.

#### 4.2.3. Ductility

It is easy to understand that the elongation of the alloy increases with an increasing in the deformation temperature, owing to the increase in the activated dislocation slip systems, and reflected by the decrease in quasi-cleavage planes and the enlarged dimples in tearing ridges [38]. When the specimens are tensile-tested at a high temperature, such as 125 °C, elongation increases in the DP region. This is because the precipitation-strengthening effect of CP regions is more robust than that of DP regions, and micro-voids are formed before the occurrence of cracks in DP regions. As is well known, the formation of micro-voids can release stress concentration, which avoids the formation of cracks and premature fracture [39].

### 4.3. Fracture Behavior

#### 4.3.1. Crack Initiation

When the AZ80A magnesium alloy is tensile-tested at room temperature, micro-cracks are easily formed in CP regions. This is attributed to the low precipitation-strengthening effect of continuous precipitates on CP regions. However, many micro-voids occur in DP regions during tensile testing at high temperatures. This is consistent with previous studies [40]. The main reason for this is the stress concentration induced by the high dislocation pile-ups in front of the elliptical or lamellar precipitates. When the micro-voids grow and connect with each other, cracks form in DP regions. With the increase in strain, the cracks grow and propagate in the materials, resulting in the fracture of the tensile specimens.

#### 4.3.2. Dependence of Temperature on Fracture Behavior

When the peak-aged specimens are subjected to tensile testing at low temperatures, the fracture length in CP regions is much longer than in DP regions. This is because when a micro-crack in the CP region grows into the boundary of the DP region, it alters its growth direction to avoid the DP region. This phenomenon is consistent with our previous argument that the precipitation-strengthening effect in DP regions is much stronger than in CP regions [28,31]. As a result, the fracture length in the CP region is much longer than that in the DP region, despite the fraction of CP and DP regions being almost equal.

At 100 °C, the DPs are softened much more conspicuously than the CPs. The percentage of fracture length in the CP and DP region corresponds to the fraction of the CP and DP region of the alloy, respectively. Moreover, the micro-cracks in DP regions can propagate into CP regions and vice versa. This indicates that the precipitation-strengthening effect of CP regions is comparable to that of DP regions.

When the alloy is tensile-tested at 175 °C, the fracture length in DP regions is much longer than in CP regions. This is because the micro-cracks tend to form and propagate in DP regions and are hard to grow into CP regions. This phenomenon also provides strong evidence for the argument that the precipitation-strengthening effect in CP regions is much better than that in DP regions at high temperatures.

## 5. Conclusions

After peak aging at 175 °C, the forged AZ80A Mg alloy achieves up to a 90% DP-region fraction. However, the solution-treated specimens at 420 °C, 450 °C, and 480 °C only obtain DP-region fractions of 58%, 48%, and 32%, respectively. After tensile testing at different temperatures, the dependence of the DP-region fraction on the mechanical properties and fracture behaviors is revealed as follows.

1. At room temperature, increasing the fraction of DP regions improves yield strength and tensile strength. However, at high temperatures, an increase in the fraction of DP regions increases the elongation but deteriorates the tensile strength. This is because the CP regions’ precipitation-strengthening effect is better than that of the DP regions at elevated temperatures.
2. During tensile testing at room temperature, micro-cracks usually initiate in CP regions, and the fracture propagation tends to penetrate the CP regions. However, micro-voids prefer to form in DP regions during tensile testing at high temperatures, contributing to a higher elongation achieved by increasing the fraction of DP regions. Moreover, the cracks propagate much more easily in DP regions than in CP regions.

## Figures and Tables

**Table 1:** Labels of the specimens after different heat treatments.

Labels of Specimens	Heat Treatment	Aging Time
T5	Aging at 175 °C	0~315 h
420T6	Solution treating at 420 °C + aging at 175 °C	0~315 h
450T6	Solution treating at 450 °C + aging at 175 °C	0~315 h
480T6	Solution treating at 480 °C + aging at 175 °C	0~315 h

**Table 2:** Room-temperature tensile mechanical properties of the AZ80A alloy with different DP-/CP-region fractions.

DP-Region Fraction	Yield Strength (MPa)	Tensile Strength (MPa)	Elongation (%)
0% (forged)	153 (±3)	289 (±3)	10.8 (±1.4)
90% (T5)	218 (±3)	327 (±2)	4.1 (±1.2)
58% (420T6)	205 (±4)	322 (±2)	5.6 (±0.5)
48% (450T6)	193 (±3)	319 (±4)	6.2 (±0.7)
32% (480T6)	185 (±3)	296 (±3)	5.4 (±0.8)

**Table 3:** High temperature mechanical properties of T6 peak-aged specimens at 175 °C after different solution treatments.

Tensile Temperature (°C)	DP-Region Fraction	Yield Strength (MPa)	Tensile Strength (MPa)	Elongation (%)
100	0% (forged)	134 (±3)	270 (±3)	20.9 (±1.8)
	90% (T5)	178 (±2)	280 (±2)	11.8 (±1.2)
	58% (420T6)	174 (±2)	281 (±3)	12.1 (±1.9)
	48% (450T6)	169 (±2)	281 (±4)	11.8 (±1.5)
	32% (480T6)	159 (±2)	273 (±4)	11.2 (±1.4)
125	0% (forged)	130 (±4)	212 (±3)	25.3 (±1.9)
	90% (T5)	169 (±3)	220 (±2)	20.7 (±1.8)
	58% (420T6)	163 (±4)	234 (±3)	16.8 (±2.3)
	48% (450T6)	157 (±3)	238 (±4)	15.4 (±2.0)
	32% (480T6)	155 (±2)	244 (±3)	13.8 (±1.8)
150	0% (forged)	124 (±4)	184 (±3)	26.4 (±2.2)
	90% (T5)	163 (±3)	189 (±3)	22.1 (±1.9)
	58% (420T6)	156 (±3)	194 (±4)	17.9 (±2.0)
	48% (450T6)	152 (±4)	200 (±3)	16.5 (±2.1)
	32% (480T6)	149 (±3)	206 (±2)	15.1 (±2.1)
175	0% (forged)	109 (±3)	156 (±3)	33.2 (±2.7)
	90% (T5)	153 (±3)	164 (±2)	24.8 (±2.0)
	58% (420T6)	150 (±2)	165 (±3)	19.9 (±2.4)
	48% (450T6)	147 (±2)	167 (±3)	18.4 (±1.8)
	32% (480T6)	145 (±4)	169 (±3)	16.7 (±1.9)

## Data Availability

All relevant data are within the paper.
